# Complete Genome Sequence of *Xanthomonas campestris* pv. *viticola* Strain CCRMXCV 80 from Brazil

**DOI:** 10.1128/genomeA.01263-17

**Published:** 2017-11-16

**Authors:** Nelson B. Lima, Marco A. S. Gama, Rosa L. R. Mariano, Wilson J. Silva, Antônio R. G. Farias, Raul M. Falcão, Lucas C. Sousa-Paula, Ana M. Benko-Iseppon, Sérgio S. L. Paiva, Valdir Q. Balbino, Elineide B. Souza

**Affiliations:** a Department of Agronomy, Federal Rural University of Pernambuco, Recife, Brazil; b Department of Genetics, Federal University of Pernambuco, Recife, Brazil; c Department of Biology, Federal Rural University of Pernambuco, Recife, Brazil

## Abstract

Here, we report the complete 5.3-Mb genome sequence of *Xanthomonas campestris* pv. *viticola* (CCRMXCV 80), which causes grapevine (*Vitis vinifera* L.) bacterial canker. Genome data will improve our understanding of the strain’s comparative genomics and epidemiology, and help to further define plant protection and quarantine procedures.

## GENOME ANNOUNCEMENT

*Xanthomonas* comprises a group of plant pathogenic bacteria that infect diverse crops around the world. *X. campestris* pv. *viticola* (Nayudu) dye is the causal agent of grapevine bacterial canker, a disease that occurs only in Brazil and India. It is a serious threat to grapevine cultivation, limiting export potential because of the effect of the disease on the quality of the fruit. Presently, it is the main grapevine bacterial disease in the northeast of Brazil, where nearly 99% of the exported grapes are produced. Additionally, *X. campestris* pv. *viticola* is considered to be a quarantine pest (A2) by the Brazilian government. The symptoms of this disease are small, dark, angular leaf spots. Later, spots may coalesce and dry up, causing necrotic areas and leaf blight. The midribs may also show discoloration. Berries show numerous depressed, dark lesions. Cankers can be observed in the petiole, stems, and rachis, as well as on bunches (1). This paper reports the whole-genome sequence of *X. campestris* pv. *viticola* strain CCRMXCV 80, which was obtained from grapevines growing in the semi-arid region of northeast Brazil. Whole-genome sequencing was performed using the Illumina HiSeq 2500 platform at the University of São Paulo’s Center for Functional Genomics. The libraries were prepared with the Illumina Nextera XT DNA library prep kit, and sequencing was performed on a HiSeq flow cell v4, with the HiSeq SBS kit v4 and 100 bp paired reads (2×). Shotgun sequencing yielded 14,817,238 read pairs. Initially, quality reads were analyzed by FastQC (2) and then treated by removing the adapters using FASTX-Toolkit (v0.0.13) (3). We used three assemblers, Abyss (v2.0.2) (4), SPAdes (v1.10) (5), and Velvet (v1.1) (6). SPAdes showed the best results, yielding 78 contigs > 500 bp (*N*_50_, 418,068 bp), with the largest contig being 973,336 bp, for a total assembly size of 5,348,596 bp with G+C content of 63.84%. The assembly statistics were generated with QUAST (v3.9) (7). The annotation using GeneMarkS (8) predicted 4,427 genes.

### Accession number(s).

This whole-genome shotgun project has been deposited at DDBJ/ENA/GenBank under the accession number NWTJ00000000. The version described in this paper is version NWTJ01000000.
